# Post Partum Hæmorrhage

**Published:** 1866-01

**Authors:** Edw’d W. Jenks

**Affiliations:** Lecturer on Diseases of Women, etc., in Detroit Preparatory School of Medicine


ART. II—*Post Partum Hæmorrhage.* By Edw'd W. Jenks, M. D., *Lecturer on Diseases of Women. etc., in Detroit Preparatory School of Medicine.*

The causes which commonly produce uterine hæmorrhage, especially that variety which attends child-birth, and the most approved methods of its treatment are already familiar to the medical profession. I have neither a new theory or a new remedy to offer, and only for the reason of a recently published article in the "Journal," which seems to deserve comment from some one, I should not attempt to write upon a subject of which so much has been said and written, and one I believe to be generally well understood.

I do not think there are many who will be mislead by the article to which I refer, but there are two classes of medical men who possibly might be. One is represented by the physician who is loose in his investigations, and who is ready to accept any new theory in regard to disease, or adopt any new remedy for its treatment that is commended in medical periodicals, unsustained by a single sound principle or true doctrine in physiology, pathology or therapeutics. The other class includes the young physician, who, just commencing practice, without having had opportunities to prove correctness of theories taught him, or lacking many of the advantages and attainments acquired only by experience and familiarity with disease, *may*, in an evil moment, adopt medical heresies and pursue useless or pernicious treatment, to the injury of his patients and the reproach of the profession.

In the November number of the "Journal published among the proceedings of the Medical Society of S. W. New York," in a paper by Dr. Irwin of Dunkirk, which seems also to have received the sanction of several members of the Society, entitled "Acetate of Lead in Uterine Hæmorrhage in heroic doses," the doses are not measured by grains, but by drachms, as the writer says, "instead of giving it in one, two or three grain doses, I give it in doses of as many drachms, and with as little danger from its use as you will meet with in your two or three grain doses, and at the same time control the hæmorrhage completely and in an instant." Dr. Irwin does not claim originality in instituting this "*heroic*" method of treatment, but attributes its origin to Dr. Joseph Workman,

Professor of Obstetrics in Victoria College, of whom he writes as follows: "In teaching the use of this remedy to his class, Dr. Workman did not attempt to explain the physiological action, but only the medicinal effects, which are those of a *direct emetic*, and an immediate powerful contraction of the uterus, thereby closing the mouths of the bleeding vessels." "The belief of the Doctor is, that it exerts a powerful influence on the sympathetic nervous center, from which you know the uterus is so freely supplied."

Prefacing the above quotation allusion is made to the agitation and distress of friends and attendants in the lying in chamber, and the extreme danger of the patient during the progress of a post partum hæmorrhage, and to some of the means used for checking the flooding as follows: "Cold is rapidly applied to the abdomen by the douche or with napkins, also to the vulva, or perhaps the tampon is applied, the position of the patient changed, the head lowered, etc., but still the hæmorrhage progresses," etc., etc.

Prominent among the objections to the treatment proposed by Dr. Irwin for uterine hæmorrhage are, its uselessness, or at least its inferiority, for it possesses not a single advantage over more rational means, and the possibility of its proving pernicious by its poisonous effects, or by depending upon it as the sole or chief agent for arresting hæmorrhage to the neglect of less objectionable, tried and better means.

Dr. I. also commends this "heroic" treatment in profuse hæmorrhage from any cause except placenta prævia, or in hydatids polypus, etc. While I consider the objection to this treatment equally great in every form of uterine hæmorrhage, yet as the Doctor's paper is principally in regard to his favorite treatment in post partum hæmorrhage, I shall not allude to the treatment of any other form of uterine hæmorrhage, but post partum, which I shall briefly consider in this connection, embodying within it my objection to the plan of treatment commended by Dr. Irwin, also giving a *resume* of more rational modes of treatment, familiar and of undoubted value.

Dr. Irwin says, "having always used the acetate of lead in my practice in doses of one to three drachms without having lost a patient by hæmorrhage from the uterus, I feel it my duty to explain and defend its use," but he gives us no opportunity to judge of the extent of his obstetric experience, or of the number of cases of post partum hæmorrhage occurring in his practice. There is many a physician with experience of years who has never lost a patient from uterine hæmorrhage. In conversation a short time since with a physician whose name is familiar to every reader of the "Journal," he informed me that during the twenty years of his practice he had not lost a patient from post partum hæmorrhage. Dr. Stebbins of this city, with a large obstetric experience during forty years of medical practice, says he has never lost a patient from any variety of uterine hæmorrhage. Other physicians have told me the same. I have never lost a patient from that cause during ten years of active medical life. Neither have I known of many deaths from that cause either in hospital or private practice. I believe if a careful estimate was to be made, that the frequency or mortality of post partum hæmorrhage would be found much greater in hospital than private practice, in crowded cities than in less densely populated towns and the country.

It has been estimated by Churchill that post partum hæmorrhage occurs once in every one hundred and twenty-two cases of child-birth, and the mortality is about one in six, so that a fatal case by this estimate will occur in between *seven and eight hundred cases of labor.* I believe even this estimate of its frequency to be a large one. Many so-called cases of post partum hæmorrhage, cease spontaneously, without the interference of art, and before designating a case as such, it is well to recollect that every case of child-birth is, or should be, attended with more or less hæmorrhage as a physiological requirement or necessary depletion whereby the distended utero placental vessels are relieved of blood, enabling the uterus to properly contract.

In hæmorrhage succeeding child-birth the blood flows from the open mouths or recently ruptured extremities of the utero placental vessels, for the uterus at this time presents the character of a recently produced wound. When the hæmorrhage continues and becomes profuse, these vessels are open, owing to the relaxation of the uterine walls, or in other words a state known among obstetricians as uterine *inertia* exists. So long as *inertia* exists flooding will continue. To avert the hæmorrhage contraction of the uterus is necessary, for with contraction the utero placental vessels are closed, and the bleeding ceases, to produce uterine contraction therefore, is the object to which all our treatment is to be directed, and that treatment only which can produce it, will prove of the least service in averting flooding Exceptional hæmorrhage may continue not as the result of uterine inertia simply, but with retained placenta or when some portion is morbidly adherent.

External flooding is easily diagnosed, as it is the only way manifested to the patient and attendants, that hæmorrhage is taking place, and from the fact of its external manifestation is the more readily treated.

Concealed or internal hæmorrhage where from some cause the os uteri is accluded by coagula or detached portion of the placenta, is of a more grave character and oftener fatal in its results. The careless or incompetent obstetrician might fail to diagnose a case of concealed hæmorrhage until too late for the exercise of human skill.

The only benefit I can conceive possible to be derived from large doses of acetate of lead, is, as acknowledged by Dr. Irwin *simply as an emetic*, for he directs a dose of ipecac to be in readiness to administer unless emesis is immediately produced. Sulphate of zinc, or any other emetic that will produce emesis will answer equally as well as the acetate of lead; the act of emesis itself brings into action the very muscles whose services are also required in aiding the expulsion of any intra-uterine substance, and in no other way does it seem possible that the acetate of lead can, as claimed, *instantly* control uterine hæmorrhage, for no remedy taken into the stomach can act as a hæmostatic until the absorbants have performed their function. Remedies acting upon the organic nerves, exciting reflex action may be instantaneous in their effects, of which we have no better example than cold and electricity. To excite the uterus to contraction during post partum hæmorrhage we cannot wait for the tardy action of remedies by absorption, neither do I consider emesis, whether produced by one remedy or another as necessary or important, for in the administration of emetics we not only add greatly to the discomfort of our patients, but if we give acetate of lead in two or three drachm doses, we administer beyond controversy poisonous doses, their only safety resting upon the powerful irritating effect of the lead upon the stomach, whereby emesis is produced.

Dr. Irwin speaks of the tampon as a means often relied upon to arrest the flooding. I know many speak highly of its merits, and rely upon it as an important aid, yet I think it will be found that they usually depend also upon other means, as grasping the uterus through the abdominal walls, thus really superseding the use of the tampon. My own opinion is that in post partum hæmorrhage the tampon possesses not a single advantage over other means, but on the contrary should never be relied upon solely, as its use is liable to produce, for obvious reasons the very thing we wish to avoid, viz: concealed hæmorrhage, and cannot produce contraction. Thus a woman may faint and die, from the uterus being distended with blood, caused by the tampon occluding the mouth of the uterus, while the physician who is relying upon it is congratulating himself upon his success in arresting the hæmorrhage.

It is needless to dwell at any length upon means (familiar to every well informed physician) as efficacious in arresting the flooding following child-birth. Every one has at his command all that in the majority of cases is necessary to stop the bleeding, and without the interference of drugs, although their administration is sometimes required to maintain permanent contraction of the uterus. Friction, pressure and cold, separately and combined, judiciously and knowingly used and applied, will generally arrest the flooding, are sufficiently "heroic," and come nearer to being instantaneous in their effects, through reflex action, than any remedy can, when taken into the stomach.

Friction upon the abdomen, or friction and pressure combined, which I will term kneading; the removal of coagula from the vagina, or possibly from the uterus, will in most instances be sufficient. If the placenta remains and the bleeding continues, let the hand be firmly but gently carried into the uterus, usually in a short time the hand and placenta will both be expelled by the uterine contraction thus produced, but they may be greatly facilitated by gently kneading the abdomen with the disengaged hand, the uterus is thus excited to contract, though reflex action by irritations, both external and external, speedily manifesting itself by the expulsion of its contents and contraction of its walls.

Cold, externally and internally, will excite speedy contraction. I prefer ice to the douche, and would recommend that before being applied externally, a piece should be put into the vagina, or even the uterus. A quantity of cold water thrown up the rectum will often prove efficacious. The only objection to the douche is the discomfort it may render to the patient before the clothing and bedding are removed. Warmth should be maintained in the extremities by the usual means. In some instances where I have maintained the uterus within my grasp through the abdominal walls, I know I have prevented further flow, which otherwise seemed inevitable, as each time I relinquished my hold, the blood would start, or the abdomen enlarge with the internal flow of blood, until the condition of the system favoring hæmorrhage, (which I shall again allude to,) was overcome. In some of these cases I have administered ergot alone, or combined with opium and brandy as the case required, and applied cold as before referred to, but continued to grasp the uterus until I considered my patients beyond danger of further hæmorrhage. In none of these cases, especially when the patients were exhausted, would I have considered vomiting advantageous, but positively pernicious. Neither do I believe that acetate of lead, in any sized doses, could act quicker or in a more efficacious manner through the "sympathetic nervous system," or as an "emetic," than the "instantaneous" reflex action excited by pressure,* friction and cold, causing the uterus to contract and the bleeding to cease.

Invaluable as ergot is, as a remedial agent, it is often injudiciously used, both in aiding child-birth and in the hæmorrhage which succeeds it. In mild cases of hæmorrhage it may be sufficient to arrest the flooding, but from the fact that it may be from ten minutes to an hour after its administration before its specific effect is apparent, it cannot be relied upon in severe cases to arrest the bleeding; yet its value is not lessened as its use *may secure permanent contraction of the uterus*, and render patients safe from renewed attacks.

We often hear physicians expressing disappointment in the use of ergot, "that it is uncertain," that "patients sometimes flow just as much," etc., etc., this uncertainty will in many cases admit of explanation, and can also in many cases be remedied. Whether

* Preventing hæmorrhage by grasping the uterus through the abdominal walls is, without doubt, in part mechanical.

ergot is given to facilitate child-birth, or for the purpose of preventing or arresting post partum hæmorrhage, in no class of patients are its desired effects more apparent and satisfactory than in women of "full plethoric habits, where the hearts are in strong action and all the vessels are gorged with blood." On the other hand distrust of its virtues and disappointment in its effects are frequent when administered to women who are weak, delicate and lax-fibred, with pale countenances, feeble pulses, spare limbs, and with weak and slow labor pains. Why is it that in one instance we see such decidedly good effects from the ergot, while in the other we observe no beneficial effects, or else those of a detrimental character? The reasons are obvious. The well known depressing influence and power of lowering the circulation, which ergot possesses, renders it peculiarly applicable, and presents no hindrance to its specific effect of securing permanent contraction of the uterus in hæmorrhage occurring among the first mentioned class of patients. But the propriety of giving ergot in cases of hemorrhage occurring among the second class is extremely doubtful, the weakened or exhausted condition of the nervous system, and enfeebled circulation, so often the producing cause of uterine hæmorrhage are increased and aggravated by its administration. In such cases the hope of stimulating the exhausted uterus to contraction is vain, as each dose tends to debilitate it still more, but if given, a full dose of opium or some one of its preparations should be given with it, as opium thus acts as both a corrugant and adjuvant. Still a better way is, to wait until the nervous energy is restored by opium before the ergot is administered.

In the treatment of post partum hæmorrhage of the last mentioned character, we possess no remedy of greater value than opium. Opium as both a stimulant and sedative, is in the physician's hands a remedy potent for evil or good, and whether given alone or as an adjuvant, should be understandingly administered without assuming that its operation must be the same, when the uterus has lost all power to contract, as when it has contracted spontaneously.

Paradoxical as it may seem, opium can cause the uterus to contract in one form of hæmorrhage, while in other cases it may cause it to relax, for its influence is manifestly different when nervous irritability is almost exhausted, than when it is excited to the highest degree. In the language of Prof. Murphy, "If nervous irritability be not impaired, or if it be increased a small dose of opium would stimulate—a larger one would exhibit 'its sedative effects; but if on the contrary that irritability is destroyed and the uterus atonic, the same large dose would only act as a stimulant. Nor will the sedative property of the medicine be observed until the nervous energy is restored. In the use of opium, therefore, strict attention should be paid to the degree of hæmorrhage and the effect upon uterine contractability. When the loss of blood is slight, or at least not sufficient to impair the tone of the uterus, a large dose would be dangerous lest it might act as a sedative, overcome the influence of the nerves and cause the uterus to relax. When the loss is great and followed by exhaustion of the uterus, then the very same quantity of the medicine will produce an opposite effect—it will act as a stimulant and cause contraction of the uterus."

The satisfactory experience I have had in the sub-dermal use of morphiæ in the treatment of various diseases, especially where a speedy effect is desired, is so great that I have no hesitancy in recommending its use by means of the hypodermic syringe in the class of hæmorrhages alluded to, where opium is particularly indicated, as patients are then more certain to experience its influence and in a much shorter space of time than when given by the stomach. I trust the day is not far distant when our list of remedies to be thus given will be much enlarged, among which the active principle of ergot would be a great desideratum. Of the prophy lactic treatment of post partum hæmorrhage much might be properly added, which I shall of necessity pass by.

In taking leave of Dr. Irwin's paper, I will simply add that it is my belief, that success in the treatment of uterine hæmorrhage can neither be secured or expected by following rules or administering remedies the same in each case, but with correct knowledge of the palpable truths of physiology, pathology and therapeutics.

---

Dr. Felix Robertson, the first white male child born in Nashville, Tenn., died in that city in June last, aged 84 years.

